# Association of metabolic syndrome with serum fibroblast growth factor 21 in kidney transplanted patients

**DOI:** 10.15171/jrip.2016.17

**Published:** 2016-04-24

**Authors:** Leila Bagheri, Maryam Hami, Mohammad-Javad Mojahedi, Mahin Ghorban Sabbagh, Hosein Ayatollahi

**Affiliations:** ^1^Kidney Transplantation Complications Research Center, Montaserieh Hospital, School of Medicine, Mashhad University of Medical Sciences, Mashhad, Iran; ^2^Hematology and Blood Banking Department, Cancer Molecular Pathology Research Center, Ghaem Hospital, School of Medicine, Mashhad University of Medical Sciences, Mashhad, Iran

**Keywords:** FGF21, Metabolic syndrome, Kidney transplantation

## Abstract

**Introduction:** Fibroblast growth factor 21 (FGF21) is a metabolic regulator with multiple beneficial effects on glucose and lipid homeostasis and insulin sensitivity.

**Objectives:** The aim of this study was to investigate the relation between the serum level of FGF21 with and metabolic syndrome (MS) in kidney transplant recipients.

**Patients and Methods:** We performed a cross-sectional study on 86 stable renal transplant recipients to detect possible relation between serum FGF21 level and MS during October 2014 and Mach 2015. Patients with past history of diabetes mellitus were excluded.

**Results:** There were 43 patients in each group with and without MS. Totally, they were 52 (60.5%) male and 34 (39.5%) female. The mean age of the MS group was significantly higher than that of non-MS group. There was not significant difference between mean serum creatinine level and glomerular filtration rate (GFR) between two groups (*P* > 0.05). The MS patients had higher weight and body mass index (BMI) (*P* < 0.05). The prevalence of BMI >25 kg/m^2^ in MS group was 25 (58.8%) versus non-MS group that only 10 (23.3%) had this condition (*P* < 0.05). The mean of FGF21 level in MS and non-MS groups was 1.23 ± 0.67 ng/l and 1.18 ± 0.71 ng/l, respectively (*P* > 0.05). There was not significant difference of serum FGF21 level between MS and non-MS patients (*P* > 0.05).

**Conclusion:** While the elevated serum FGF21 level was found in subjects with insulin resistant states, however, this study revealed that serum FGF21 levels were not significantly increased in renal transplanted recipients with MS as compared with non-MS group.

**Implication for health policy/practice/research/medical education:**

In a cross-sectional study on 86 stable renal transplant recipients to detect the association between serum fibroblast growth factor 21 (FGF21) level with metabolic syndrome (MS), we found serum FGF21 levels were not significantly raised in the renal transplant recipients with MS group when compared with non-MS group.

## Introduction

The fibroblast growth factor (FGF) family is composed of 22 members with a wide range of biological functions including cell growth, development, angiogenesis, and wound healing (1-5). Human FGF21 is a polypeptide of 181 amino acids produced predominantly by the liver (3). However, it can be secreted by other tissues involved in glucose and lipid metabolism such as the adipose tissue, pancreas and skeletal muscle (6). It has been shown that this factor possesses potent beneficial effects on glucose and lipid metabolism and insulin sensitivity in animal models (7,8). The multiple beneficial effects of FGF21 on glucose and lipid metabolism and insulin sensitivity suggest that it might represent a promising therapeutic agent to treat diabetes and other obesity-related metabolic disorders (9,10).

However, high serum FGF21 levels are found in obese subjects (11,12), it can be increased in subjects with other insulin resistant states such as dyslipidemia and coronary artery disease (13) non-alcoholic fatty liver disease (NAFLD) and polycystic ovarian syndrome (10-16).

In general, population with the metabolic syndrome (MS) is a group of linked metabolic abnormalities that is strongly associated with the development of atherosclerotic cardiovascular disease (CVD) (17-19).

Based on the previous studies, the prevalence of MS in renal transplant recipients was higher than general population, that it can be explained by effects of immunosuppressive therapy consisted of diabetogenic drugs such as corticosteroids and tacrolimus (20). In this group of patients, MS has been shown to be an independent risk factor for chronic allograft dysfunction (CAD), graft failure, new-onset diabetes, and CVD (21). It is approved that CVD is a cause of mortality in the renal transplant recipients (22) and obesity-related hyperfiltration might participate in proteinuria and accelerate graft loss (23), as more than threefold increases in graft loss has been reported in patients with MS (21).

These data, show the importance of early diagnosis of MS and its prevention, which can lead to higher survival of this group of patients as well as their graft survival.

There are two diagnostic criteria for MS. First, the National Cholesterol Education Program (NCEP) Adult Treatment Panel III (ATP III) criteria (24), and the second is the International Diabetes Foundation (IDF) criteria (25).

MS can be defined by ATP III criteria, even in the absence of central obesity (26). Thus, it has been used in the most previous studies in the renal transplant recipient population (27,28).

## Objectives

The aim of this study was to evaluate the association of serum FGF21 level and lipid metabolism and its regulation in kidney transplant recipients. We also compared its serum level based on graft function.

## Patients and Methods

### Study population

A total of 176 renal transplant recipients from the outpatient transplant clinics at the Montaserieh hospital of Mashhad University of Medical Sciences were evaluated. The inclusion criteria was consisted of age more than 18 years old, kidney transplantation period at least 6 months, stable graft function (serum creatinine level less than 2 mg/dl) in last 3 months. Finally 86 patients (52 men and 34 women) entered to study. This cross-sectional study was conducted between October 2014 and March 2015.

### Data collection

All recipients were on triple immunosuppressive therapy with tacrolimus or cyclosporine, azathioprine or mycophenolate mofetil and prednisolone. As all were more than 6 months post-transplant, they were taking the maintenance dose of steroids (2.5-5 mg/day of prednisolone).

Transplant recipients were stratified into two categories with or without MS by modified NCEP- ATP III criteria for Asians (Iranian modified): serum TG >150 mg/dl or specific treatment for this lipid abnormality, serum high-density lipoprotein cholesterol (HDL-C) <40 mg/dl in men or <50 mg/dl in women or specific treatment for this lipid abnormality, systolic blood pressure (SBP) ≥130 mm Hg and diastolic blood pressure (DBP) ≥ 85 mm Hg or use of antihypertensive medication, fasting blood sugar (FBS) >100 mg/dl or use of antidiabetic medication, waist girth ≥92 cm in both gender.

Patients who had three or more risk factors were accepted to have MS group and reminders who had two or less risk factor were categorized as not having MS (non-MS group).

Patients after 12-hour fasting were admitted to get blood sample to check serum creatinine, triglycerides, HDL-C and plasma glucose concentrations. Blood pressure was reported as the average of two measurements taken at 5-minute interval. Hypertension was defined by taking antihypertensive drugs and/or SBP more than 130 mm Hg or DBP more than 85 mm Hg. Body mass index (BMI) was calculated by the formula weight/height^2^ as kg/m^2^. Waist circumference was measured midway between the iliac crest and costal margins.

Human FGF21 enzyme-linked immunosorbent assay (ELISA) kits were obtained from Abcom Company. The assay was conducted according to the manufacturer’s protocol. A calibration curve was constructed by plotting the absorbance values at 450 nm versus the FGF21 concentrations of the calibrators, and concentrations of unknown samples were determined using this calibration curve. The intra- and inter-assay variations were 4.7% and 7.2%, respectively. We obtained 5 cc venous blood samples from each subject. Then, serum FGF21 levels were evaluated on these samples.

### Exclusion criteria

Patients with pre-transplant diabetes mellitus and post-transplant diabetes mellitus, overt infection, on immunosuppressive drugs of the target organ inhibitor group as rapamycin (sirolimus) were excluded.

### Ethical issues

The research followed the tenets of the Declaration of Helsinki. The nature of the study was explained to the participants and they enrolled their satisfactions. They were free to leave the study at any time. The research was approved by the Ethical Committee of Mashhad University of Medical Sciences, Mashhad, Iran.

### Statistical analysis

The data was analyzed by SPSS version 20.0. All data were presented as the mean ± standard deviation (SD). Differences between groups were compared with independent *t* test. Chi-square test was used to compare categorical variables. Pearson correlation was applied as appropriate for comparisons between quantitative variables. *P* value less than 0.05 was considered statistically significant.

## Results

Using modified (Asian) NCEP ATP III criteria, a total of 43 (50%) of 86 was enrolled to the study (renal transplanted patients who had MS). Demographic and transplant characteristics of patients with or without MS were shown in Table 1. As shown, the mean age of MS-group was higher than that of non-MS group (*P*<0.05), and BMI of patients with MS was significantly more than that of non-MS patients (*P*<0.05). Other factors such as gender, type of donor, dialysis duration, graft function were similar in both groups (*P*>0.05).

**Table 1:** Demographic and transplant characteristics of patients with/without metabolic syndrome

	**Without MS (n = 43)**	**With MS (n = 43)**	***P*** ** value**
Age (years)	34.63±11.02	40.16±10.19	0.01
M/F	22/21	30/13	0.078
Donor (C/A)	24/19	24/19	1.0
Dialysis (HD/PD)	41/2	41/2	1.0
Dialysis duration (months)	28.12±22.64	27.63±13.83	0.074
Weight (kg)	59.96±12.61	71.19±12.24	0.001
BMI (kg/m^2^)	22.58±3.64	25.30±3.84	0.001
Serum creatinine (mg/dl)	1.30±0.32	1.39±0.31	0.789
eGFR (MDRD)	62.90±18.88	59.39±19.21	0.395

Abbreviations: BMI, Body mass index; eGFR, estimated glomerular filtration rate

Comparison between components of MS criteria in the both patient groups was shown in Table 2. It is obvious that there is significant difference between each component of (Asian) NCEP ATP III criteria in two groups. Frequency of hypertension was significantly higher in MS group rather than non-MS group (*P*<0.000); as 40 (74.1%) of MS group had hypertension, whereas 3 (9.4%) in non-MS group suffered of it.

**Table 2:** The mean of components of (Asian) NCEP ATP III criteria

	**Without MS (n = 50)**	**With MS (n = 56)**	***P*** ** value**
Serum TG (mg/dl)	112.56±39.95	190.55±78.75	0.000
Serum HDL (mg/dl)	48.00±10.95	40.67±12.14	0.004
FBS (mg/dl)	85.86±9.60	94.00±13.08	0.001
SBP (mm Hg)	118.26±11.38	130.58±11.50	0.000
DBP (mm Hg)	74.88±9.35	83.37±9.61	0.000
Waist girth (cm)	80.69±9.01	93.39±10.88	0.000

Abbreviations: HDL, high-density lipoprotein; SBP, systolic blood pressure; DBP, diastolic blood pressure; TG, triglyceride; FBS, fasting blood sugar.

**Figure 1:**
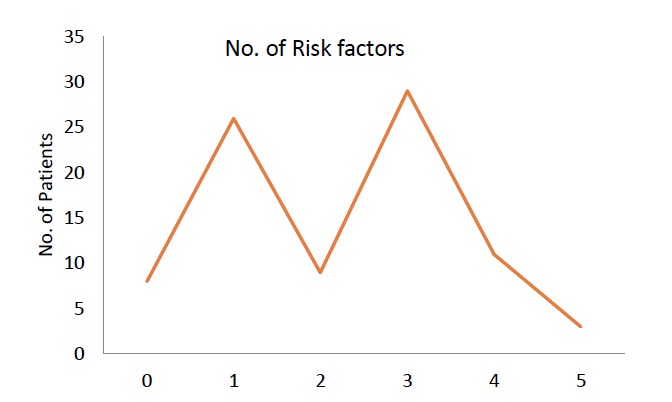
Frequency of MS in our patients by modified NCEP ATP III criteria based on the number of risk factors.

The frequency of risk factors of (Asian) NCEP ATP III criteria in our patients was shown in Figure 1.

Serum FGF21 levels were evaluated in all of patients. The means of FGF21 levels in MS and non-MS groups were 1.23±0.67 ng/l and 1.18±0.71 ng/l, respectively (*P*>0.05).

The means of serum FGF21 levels were compared in living donors and deceased donor kidney transplant recipients. There was no significant difference between them (living donor: 1.33±0.61 ng/l versus deceased donor: 1.11±0.72 ng/l, *P*=0.137). Furthermore, there was not significant correlation between serum FGF21 level and frequency of kidney transplantation (first transplant [n**=83, 1.21±0.69 ng/l versus twice [n**=3], 1.23±0.71 ng/l, *P*=0.86), type of dialysis (HD [n**=82], 1.21±0.69 ng/l versus PD [n**=4], 1.30±0.41 ng/l, *P*=0.81).

**Figure 2:**
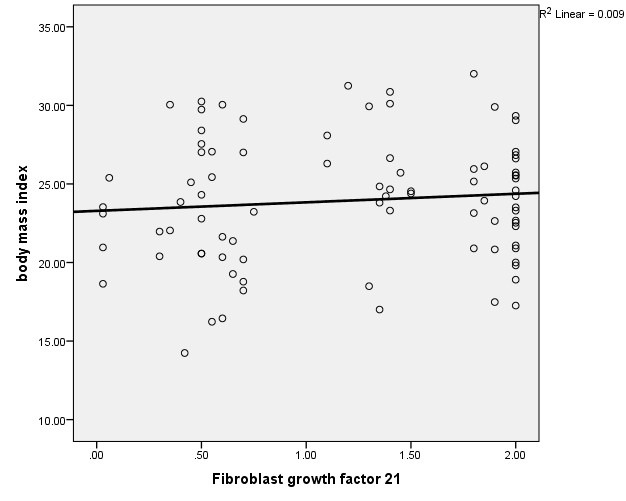
Correlation between FGF21 level and BMI that was normally distributed.

**Figure 3:**
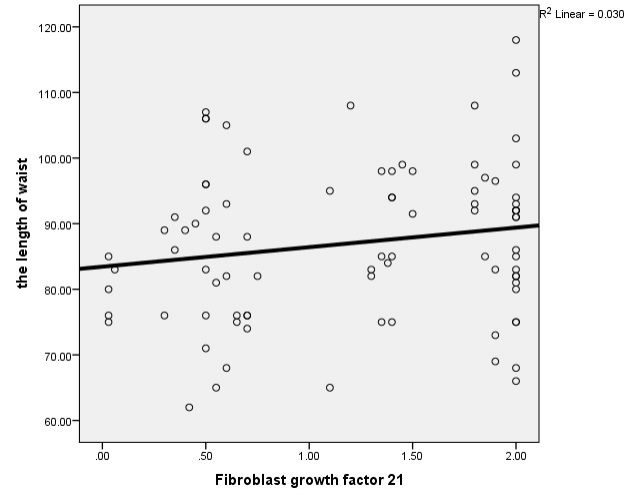
Correlation between FGF21 level and waist circumference that was normally distributed.

We investigated the relation between serum FGF21 levels and anthropometric parameters. This analysis showed no significant positive association among serum FGF21 level and BMI (Figure 2), and waist circumference, (Figure 3; *P*>0.05). The correlation between FGF21 level and cardio-metabolic risk factors were evaluated. Serum FGF21 level was positively correlated with FBS, HDL-C, duration of dialysis and was negatively associated with serum triglyceride and uric acid. However, these correlations were not statistically significant (Table 3).

**Table 3:** Correlations of serum FGF21 level with cardio-metabolic risk factors

	**r**	***P***
Age	0.15	0.17
BMI	0.09	0.39
Waist circumference	0.14	0.21
FBS	0.17	0.11
TG	-0.09	0.43
HDL cholesterol	0.07	0.50
SBP	0.1	0.37
DBP	0.11	0.28
Uric acid	-0.04	0.71
Dialysis duration	0.02	0.84

Abbreviations: HDL, high-density lipoprotein; SBP, systolic blood pressure; DBP, diastolic blood pressure; TG, triglyceride; FBS, fasting blood sugar; BMI, Body mass index

The frequency of MS were checked by original NCEP ATPIII criteria in all patients and then compared serum FGF21 levels in them. There was no significant difference between MS and non-MS groups ([n**=35], 1.21±0.66 ng/l versus [n**=51], 1.20±0.70 ng/l, *P*=0.989).

## Discussion

The prevalence of MS in the general population varies widely among ethnic background and also according to the criteria to define it. It sounds proportion of people with MS is totally growing because of the increase in obesity and aging in population in last decades (29,30).

The prevalence of the MS in dialysis patients is more than general population and it may be higher after transplantation due to weight gain and the detrimental metabolic effects of immunosuppressive drugs (29).

Our study revealed that patients with MS were significantly older than others. Although it was predictable, as there are similar results in general population. In the United States, the overall prevalence of MS in general population was 22%, with an age-dependent increase (6.7%, 43.5%, and 42.0% for ages 20 to 29, 60 to 69, and >70 years, respectively) (29).

Although there are some reports in general population such as Grundy that showed MS is more common in men than women in Greece (31), another study in the United States and Canada reported that MS affected both sex equally (29). We did not find any significant difference among patients based on gender, it may be due to lower sample size and also we evaluated this syndrome in a specific subgroup of people.

The calcineurin inhibitors consist of cyclosporine and tacrolimus were used as main drugs in immunosuppressive regimens in our patients. Both of them are associated with unfavorable effects on lipid level, blood pressure, glucose metabolism, and graft function (32,33). In our study, drug levels in all patients were in therapeutic ranges.

Corticosteroids, a mainstay of most regimens despite aggressive efforts on weaning, are associated with significant elevation in cardiovascular risk (34), and with increase in the risk of MS in renal transplant recipients. It is used with high doses in the first months of transplantation, thus patients entered to study that received their grafts at least 6 months ago. At this time, all of them administered prednisolone between 2.5 to 10 mg/day.

The MS in transplant recipients has numerous detrimental effects such as the increased risk of new onset diabetes, CVD events and patient death. In addition, it has also been linked with the accelerated loss of graft function, proteinuria and ultimately graft loss (35). In our study, we selected patients with stable renal function and both of two groups had acceptable graft function, which was in an agreement with Nafar et al (36). They reported, graft function was stable in both groups after 12-month following up of their patients (36).

Hence, it is appeared that MS affects on grafts probably during a long period. Thus it seems that it needs longer follow up of patients.

Fibroblast growth factor 21 (FGF21), a metabolic hormone predominantly produced by the liver, is also expressed in the adipocytes and the pancreas. It regulates glucose and lipid metabolism through pleiotropic actions in these tissues and the brain. In mice, fasting leads to the increased PPAR-a mediated expression of FGF21 in the liver where it stimulates gluconeogenesis, fatty acid oxidation, and ketogenesis, as an adaptive response to fasting and starvation. In the fed state, FGF21 acts as an autocrine factor in adipocytes regulating the activity of peroxisome proliferator-activated receptors (PPAR)-alpha through a feedforward loop mechanism. Administration of recombinant FGF21 has been shown to confer multiple metabolic benefits on insulin sensitivity, blood glucose, lipid profile and body weight in the obese mice and diabetic monkeys without mitogenic or other side effects. Such findings highlight the potential role of FGF21 as a therapeutic agent for obesity-related medical conditions (37).

However, high circulating FGF21 levels are found in obesity and it is related to cardio-metabolic disorders including the MS, type 2 diabetes, NAFLD and coronary artery disease in human studies (37). These findings may indicate the presence of FGF21 resistance or compensatory responses to the underlying metabolic stress, and imply the need for supra-physiological doses of FGF21 to achieve therapeutic efficacy. On the other hand, serum FGF21 level has been implicated as a potential biomarker for the early detection of these cardio-metabolic disorders (37).

In this study, we investigated the correlation of serum FGF21 levels with MS in renal transplanted subjects. We observed no significant differences in serum FGF21 levels between MS and non-MS groups even with history of hemodialysis or peritoneal dialysis before transplantation, living donor or deceased donor recipients. There were no significant correlations between FGF21 level and sex, age, BMI, waist circumference, TG, HDL cholesterol, FBS, uric acid, duration of dialysis before transplantation and frequency of transplantation. However, Kosola et al (38) evaluated serum FGF21 level in pediatric liver transplant patients. They revealed higher FGF21 level in patients than controls. While the study was not on renal transplant subject, thus this difference may be due to some metabolic differences in liver versus kidney transplant recipients.

## Conclusion

While the raised serum FGF21 levels were found in subjects with insulin resistant states, the present study revealed serum FGF21 level was not significantly elevated in the renal transplant recipients with MS group as compared with non-MS group. We suggest more studies in this view in kidney transplant population with larger sample size.

## Limitations of the study

There are some limitations in our study including a relatively small sample size. In addition, study design was cross-sectional and did not address the cause-effect relation between serum FGF21 level and MS. Further prospective studies in different ethnic groups with higher sample size could help to find the association of serum FGF21 with MS and its cardiovascular complications.

## Authors’ contribution

LB; acquisition of data and analysis and interpretation of them. MH; conception and design, drafting the article and revising it. MJM; final approval of the version. MGS; acquisition of data. HA; analysis and interpretation of data.

## Conflicts of interest

The authors declared no competing interests.

## Ethical considerations

Ethical issues (including plagiarism, data fabrication, double publication) have been completely observed by the authors.

## Funding/ Support

This paper is extracted from nephrology fellowship thesis of Leila Bagheri (Thesis number # T-3376) and financial support was provided by the research vice-chancellor of Mashhad University of Medical Sciences.
